# The promotion function of Berberine for osteogenic differentiation of human periodontal ligament stem cells via ERK-FOS pathway mediated by EGFR

**DOI:** 10.1038/s41598-018-21116-3

**Published:** 2018-02-12

**Authors:** Jin Liu, Xiaodan Zhao, Dandan Pei, Guo Sun, Ye Li, Chunhui Zhu, Cui Qiang, Junyi Sun, Jianfeng Shi, Yan Dong, Jianzhong Gou, Sicen Wang, Ang Li

**Affiliations:** 1 Key Laboratory of Shannxi Province for Craniofacial Precision Medicine Research, College of Stomatology, Xi’an Jiaotong University, 98 XiWu Road, Xi’an, Shannxi 710004 People’s Republic of China; 2 Clinical Research Center of Shannxi Province for Dental and Maxillofacial Diseases, College of Stomatology, Xi’an Jiaotong University, 98 XiWu Road, Xi’an, Shannxi 710004 People’s Republic of China; 3 Department of Periodontology, College of Stomatology, Xi’an Jiaotong University, 98 XiWu Road, Xi’an, Shannxi 710004 People’s Republic of China; 4 Department of Prothodontics, College of Stomatology, Xi’an Jiaotong University, 98 XiWu Road, Xi’an, Shannxi 710004 People’s Republic of China; 5 Research Center of Stomatology, College of Stomatology, Xi’an Jiaotong University, 98 XiWu Road, Xi’an, Shannxi 710004 People’s Republic of China; 6 The Second Affiliated Hospital, Xi’an Jiaotong University, 157 XiWu Road, Xi’an, Shannxi 710004 People’s Republic of China; 7 School of Pharmacy, Xi’an Jiaotong University, 76 Yanta West Road, Xi’an, 710 061 Shannxi People’s Republic of China

## Abstract

*Coptidis Rhizoma* binds to the membrane receptors on hPDLSC/CMC, and the active ingredient Berberine (BER) that can be extracted from it may promote the proliferation and osteogenesis of periodontal ligament stem cells (hPDLSC). The membrane receptor that binds with BER on the cell surface of hPDLSC, the mechanism of direct interaction between BER and hPDLSC, and the related signal pathway are not yet clear. In this research, EGFR was screened as the affinity membrane receptor between BER and hPDLSC, through retention on CMC, competition with BER and by using a molecular docking simulation score. At the same time, the MAPK PCR Array was selected to screen the target genes that changed when hPDLSC was simulated by BER. In conclusion, BER may bind to EGFR on the cell membrane of hPDLSC so the intracellular ERK signalling pathways activate, and nuclear-related genes of FOS change, resulting in the effect of osteogenesis on PDLSC.

## Introduction

The periodontal ligament (PDL) is a special connective tissue located between the alveolar bone and the tooth cementum. Human periodontal ligament stem cells (hPDLSC) are an important cell population with a multi-directional differentiation potential in the PDL; they have a dynamic role in maintaining periodontal homeostasis and are responsible for remodelling and regeneration of periodontal tissues^1,2^. Currently, regenerative measures to restore periodontal tissue are the ultimate goal of treatment for chronic periodontitis. For these reasons, hPDLSC are considered the most important target cells for the treatment of periodontitis.

Traditional Chinese medicine (TCM) has been praised in the world of medicine due to its effects in promoting cell proliferation, regulating bone metabolism, etc.^3,4^. Chronic periodontitis can lead to the damage and the destruction of periodontal support tissue. The goal in treating this disease is to achieve the regeneration and reconstruction of periodontal tissue, especially periodontal bone tissue. Therefore, TCM is very suitable for the treatment of chronic periodontitis. *Coptidis Rhizoma* can be used to control inflammation of chronic periodontitis and inhibition of alveolar bone resorption, with small side effects. Berberine (BER) is the main drug component in *Coptidis Rhizoma*. However, in previous research, most TCM were studied as a compound preparation. Also, the compositions were complex and the mechanism not clear. With the development of various drug purification and analytical techniques, the active ingredients in TCM (such as Artemisinin) have been gradually separated and described by in-depth studies. This has aroused increased attention from many scholars: that the active ingredients have a good effect in a variety of diseases.

The identification of active components in natural sources is very difficult. In this regard, cell membrane chromatography (CMC), a novel technique based on biological affinity chromatography, may be a viable approach, as it is effective in separating ingredients that are active toward specific membrane receptors. According to the literature^5,6^, through the CMC system established by cells highly expressing the specific receptor, the active ingredients binding with this specific receptor can be selected from TCM and its biologically effect validated for treating diseases. In early research^7^, using CMC with an online HPLC/MS system, it was found that *Coptidis Rhizoma* could bind to the membrane receptors on the hPDLSC/CMC, and that the active ingredient Berberine extracted from *Coptidis Rhizoma* could promote the proliferation and osteogenesis of hPDLSC. Because hPDLSC used in our study were primary cultured from PDL, there were a variety of membrane receptors existing in the surfaces of cells. It was not clear which cell membrane receptor was bound with the drug ligand from *Coptidis Rhizoma* and BER and the correlation effect between BER and hPDLSC and its related signal pathway has not been reported. The role of BER on the other cells^8^, and other drugs on hPDLSC^9^, are both related to the MAPK signalling pathway. At the same time, it is also reported that the MAPK signalling pathway plays an important role in the osteogenesis of cells^10^.

In this research, we propose the following hypothesis: that BER may bind to a specific receptor on the surface of the cell membrane of hPDLSC so the intracellular signalling pathway is subsequently activated, then the nuclear-related genes changed until the osteogenesis effect of hPDLSC is finally regulated.

Through the method of cell membrane activity screening, we attempted to find the target sites for BER binding to hPDLSC and the related mechanism to promote osteogenesis, in order to provide an experimental basis for the development of TCM for the treatment of periodontal bone destruction.

## Results

### BER promotes hPDLSC osteogenesis in the early, middle and late stage

**Figure 1:**
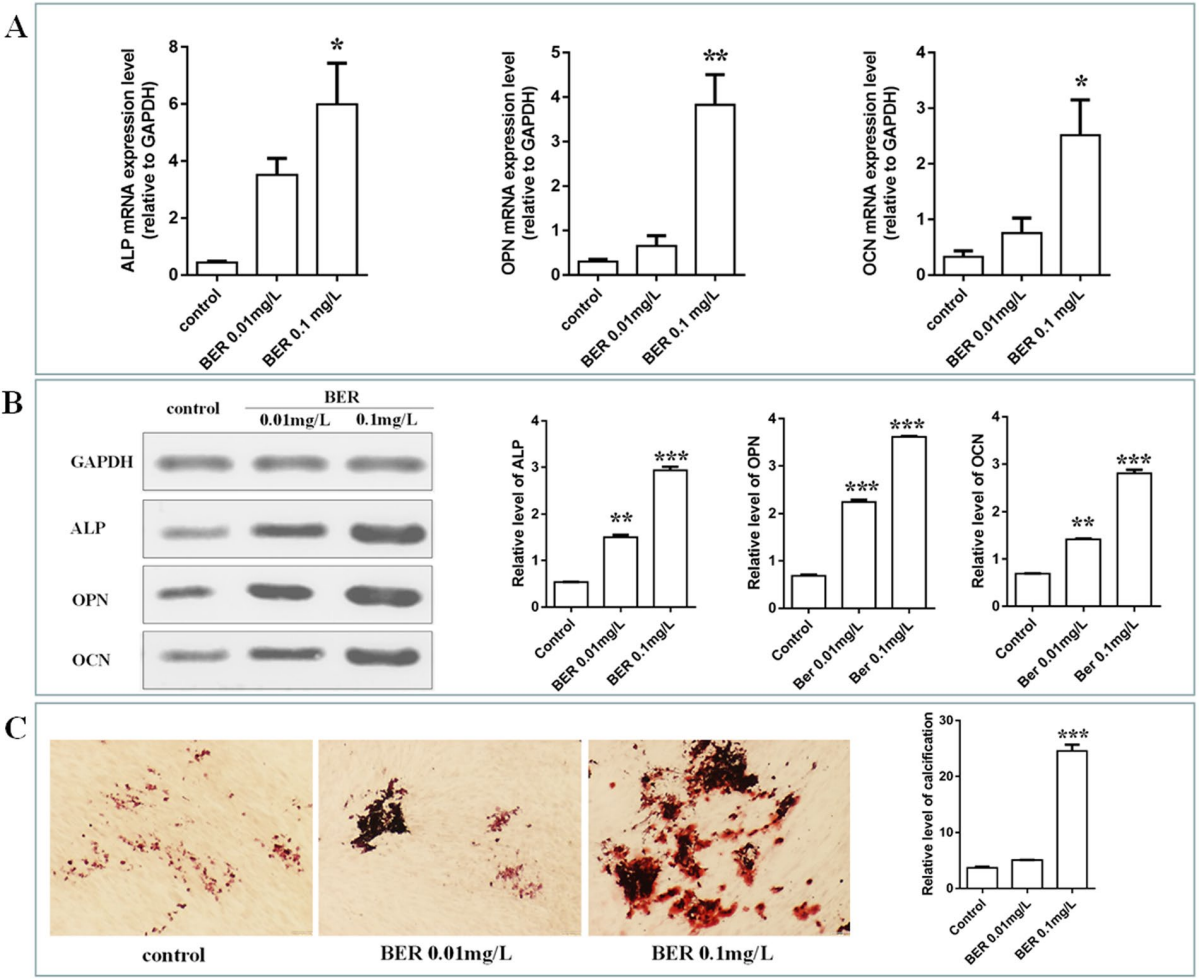
Effect of BER on osteogenesis differentiation of hPDLSC. The expression of ALP, OPN, OCN in control, BER 0.01 and 0.1 mg/L for 15 min were examined using RT-PCR (**A**) and western blot (**B**). (*vs* control, *P < 0.05; **P < 0.01, ***P < 0.001); The effect of BER on osteogenesis in osteoblast-induced conditions, which were stained with alizarin red (**C**). (BER: Berberine).

To verify the osteogenesis influence of BER on hPDLSC, different concentrations of BER (0.01 and 0.1 mg/L) were introduced into the cells. ALP activity is a well-established marker for early osteogenic differentiation at day^11^, and its transcriptional and translation activity level was significantly increased in the BER-treated group compared to the control (Fig. 1A and B), especially in the 0.1 mg/L group. These results suggest that BER promoted early osteogenic differentiation of hPDLSC. To further investigate the ability of BER to promote hPDLSC osteogenic differentiation, the expression of osteogenesis differentiation-related genes (the middle and late stages in the osteogenesis differentiation period) was investigated at 14 days post BER stimulation. As anticipated, the expression levels of OPN and OCN were significantly higher than those in the control group (Fig. 1A and B). Taken together, these observations confirmed the ability of 0.1 mg/L BER to promote early, intermediate and late bone differentiation of hPDLSC. At the same time, the calcified nodules were stained with alizarin red, which indicated BER could promote the deposition and calcification of extracorporeal calcification (Fig. 1C).

### Screening EGFR as the possible membrane receptor of BER activity binding to the hPDLSC

**Figure 2:**
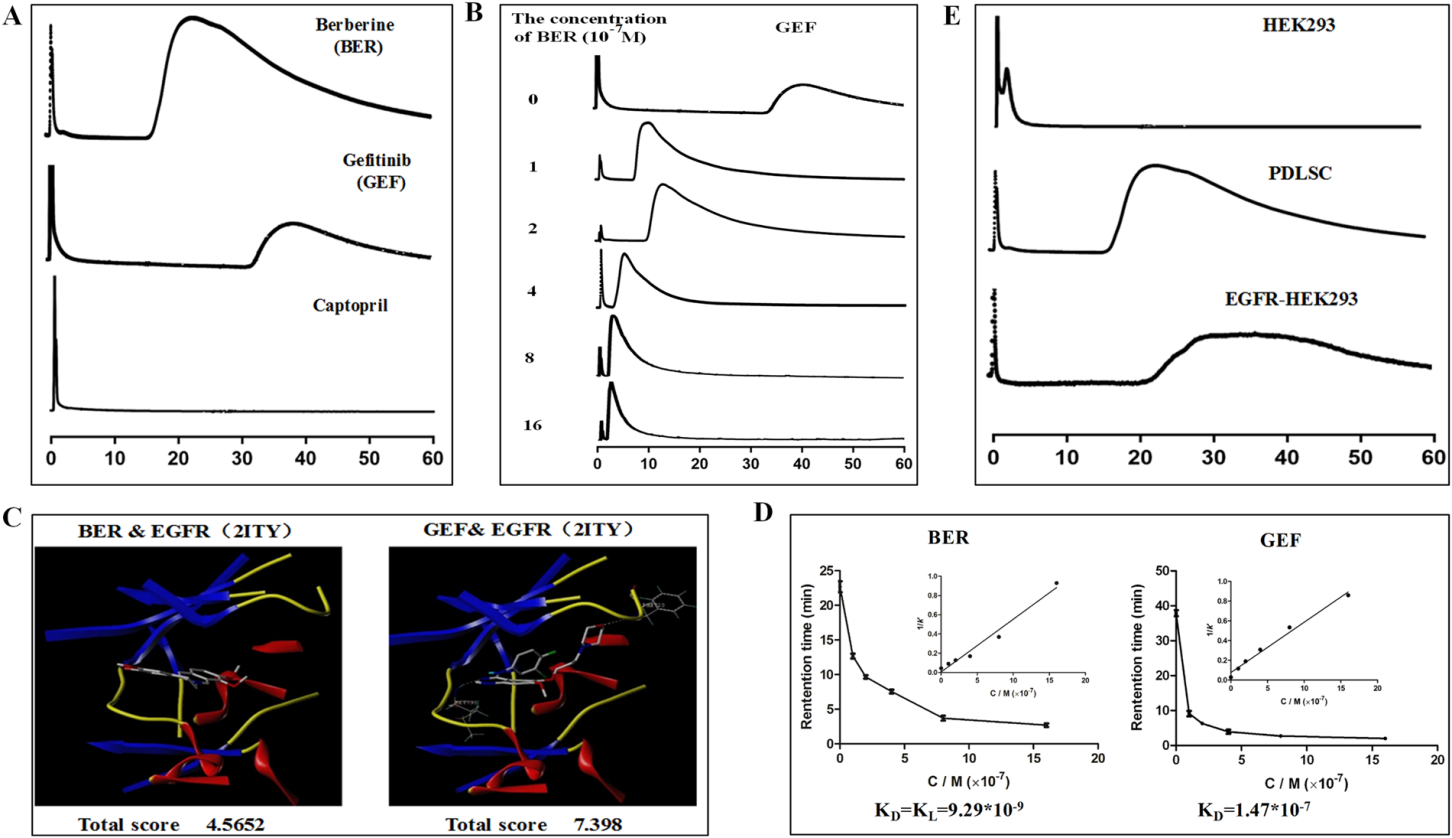
Screening the possible membrane receptor of BER activity binding to the hPDLSC. The situation of retention for different receptor antagonists (**A**) and competitions with BER on hPDLSC-CMC (**B**); Molecular docking experiments and interactions between Gefitinib and BER with cell membrane receptor (**C,D**); The retention times of BER in different CMC established with 3 cells (HEK293, hPDLSC and EGFR-HEK293) (**E**).

hPDLSC-CMC was established using cultured hPDLSC and the establishment method and system stability detection were detected as shown in the literature^7^. BER and different membrane receptor inhibitors (Gefitinib, Captopril and others) passed through the hPDLSC/CMC system; BER and Gefitinib (GEF) was retained; but Captopril (CAP) had no retained components (Fig. 2A.). It was suggested that GEF, the receptor inhibitors for epidermal growth factor receptors (EGFR), could bind to the hPDLSC through their specific membrane receptors. The retention time of GEF on this CMC was continuously decreased with an increasing concentration of BER (from 0 to16 × 10^−7^ mol/L) in the mobile phase (Fig. 2B). This illustrated that GEF and BER may have the same membrane receptor on the hPDLSC cell surface. In other words, BER may be binding with the hPDLSC through the EGFR.

The scores for the molecular docking (Fig. 2C) between BER&EGFR and GEF&EGFR were S_BER&EGFR (2ITY)_ = 4.5652 and S_GEF&EGFR (2ITY)_ = 7.398. These results suggested that BER and EGFR have a binding activity. The corresponding plot of 1/k′ versus [L]m is presented in Fig. 2D. The equilibrium dissociation constants obtained were K_BER_ = 9.29 * 10^−9^ and K_GEF_ = 1.47 * 10^−7^. Competitive binding capacity, molecular docking, and equilibrium dissociation constants assessed that GEF and BER both had the ability to bind to the hPDLSC. The results illustrate that BER would primarily associate with EGFR on the surface of the hPDLSC cell membrane, thereby enabling the BER to remain active on the CMC. Simultaneously, hPDLSC and EGFR-HEK293 had a relatively high expression of EGFR in comparison with HEK293, while EGFR-HEK293 had the highest expression. The retention time of BER in EGFR high-level expression cell lines was significantly prolonged in different CMC establishments by the three cells, which indicated that BER had the best affinity with the membrane receptor when enhanced with EGFR (EGFR-HEK293) (Fig. 2E).

### Effects of BER on MAPK/ERK signalling pathway

**Figure 3:**
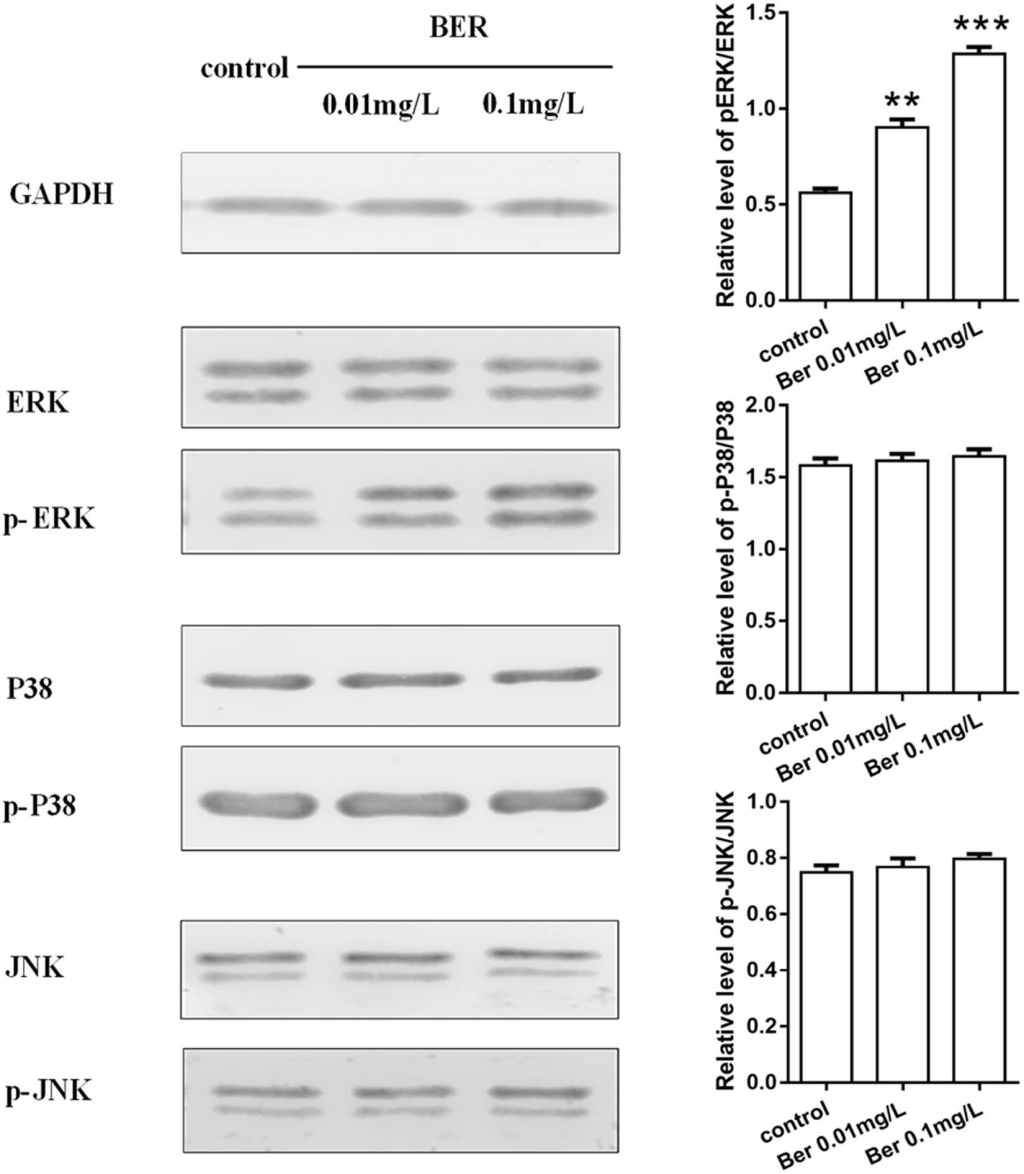
Effects of BER on the MAPK/ERK signalling pathway. Western blotting results for detection of ERK, p-ERK, P38, p-P38 and JNK, p-JNK secreted by hPDLSC cultured on different samples (control, BER 0.01 and 0.1 mg/L) for 15 min (*vs* control, *P < 0.05; **P < 0.01, ***P < 0.001) (BER: Berberine).

After 15 min incubation with 0.01 and 0.1 mg/L BER (Fig. 3), a noticeable increase of p-ERK expression of hPDLSC was shown on BER, especially with 0.1 mg/L BER, while the protein level of p-P38 and pJNK remained low in all groups. These results indicate activation of the MAPK/ERK signalling pathway in hPDLSC cultured on 0.1 mg/L BER, while the other signalling pathways were not seen.

### Screening FOS as the target gene with MAPK-PCR chip

**Figure 4:**
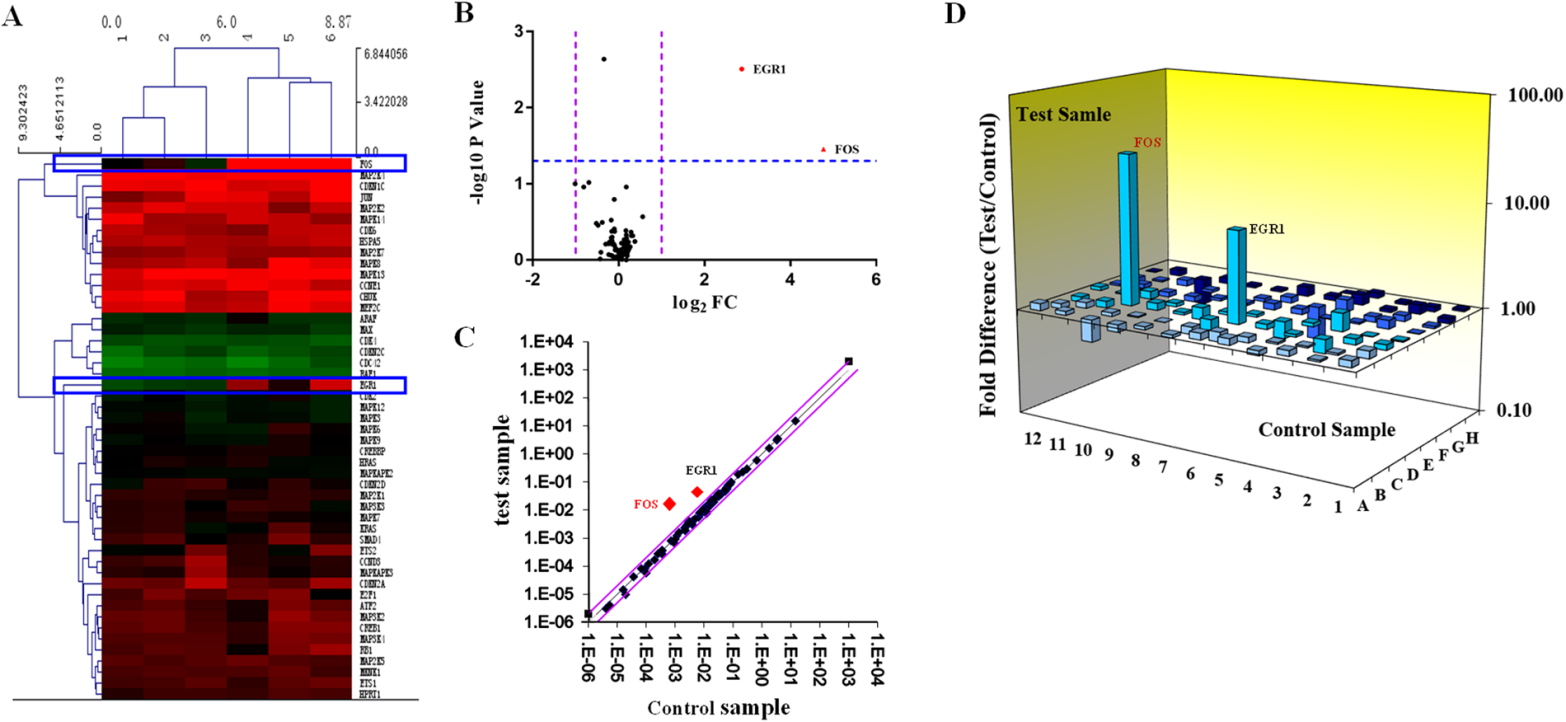
Screening FOS as the target gene with MAPK-PCR chip. After intervention with 0.1 mg/L BER 15 mins, the results of MAPK-PCR chip are as follows: (**A**) Clustering map, (**B**) volcano plot, (**C**) scatter plot, (**D**) 3D profile.

After intervention with 0.1 mg/L BER 15 mins, the RNA of hPDLSC was extracted and subjected to MAPK signalling PCR chip detection. The results showed two significantly elevated genes: FOS and EGR1 (Fig. 4). They were 27 times and 7 times higher than the control group, respectively. It was suggested that two essential genes in the hPDLSC could be increased significantly by BER. We next selected FOS (the most significantly changed gene) as the target gene to study BER in promoting cell proliferation and osteogenesis.

### BER affinity to hPDLSC through EGFR-ERK-FOS

**Figure 5:**
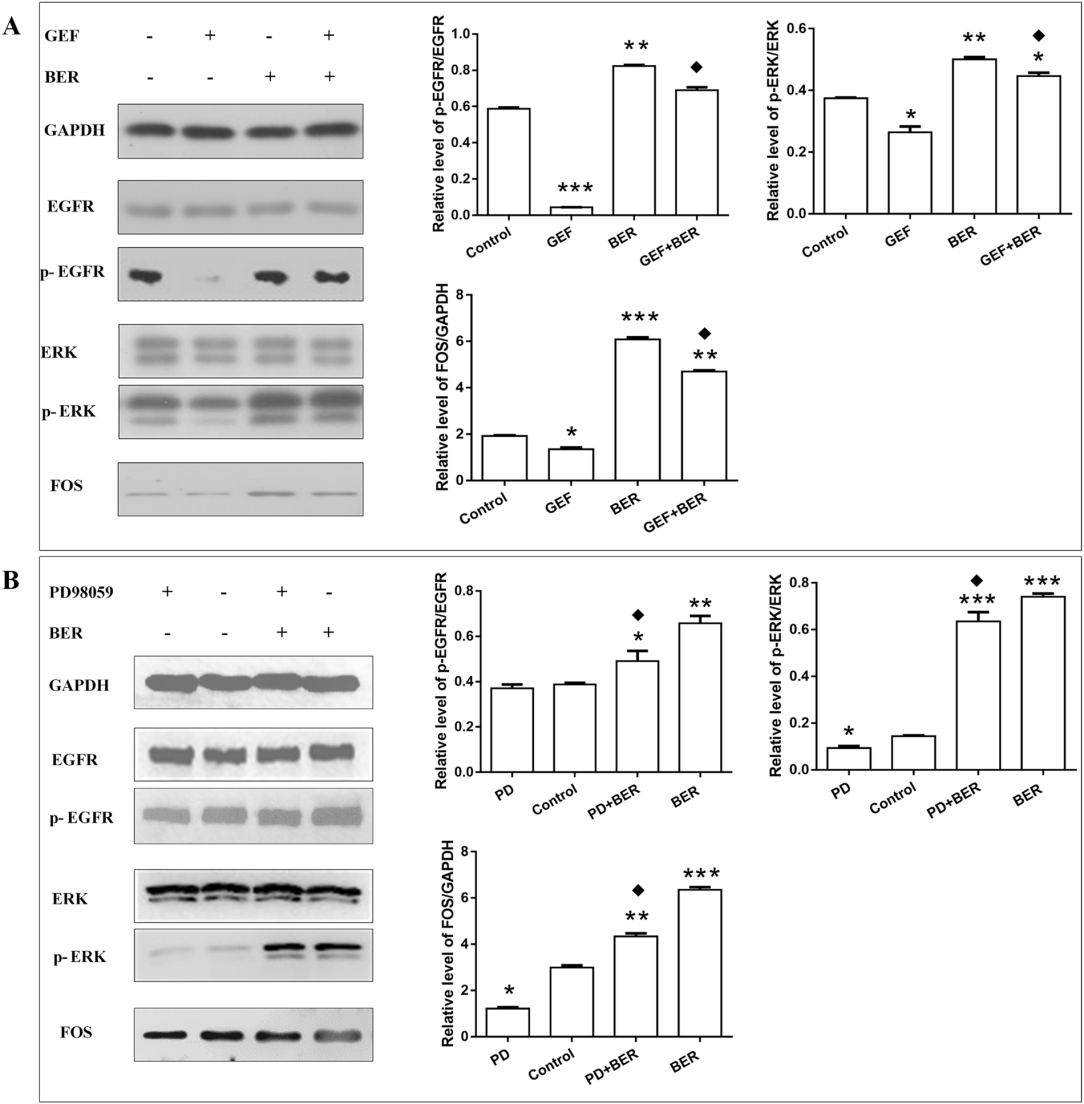
The effects of affinity between BER and hPDLSC through EGFR-ERK-FOS. (**A**) The expression of EGFR, p-EGFR, ERK, p-ERK and FOS in four groups (from left to right: control; 2 μg/L GEF 24 h; 0.1 mg/L BER 15 min; pretreatment with 2 μg/L GEF 24 h, then intervened with 0.1 mg/L BER for 15 min) were examined using western blot. (**B**) The expression of EGFR, p-EGFR, ERK, p-ERK and FOS in four groups (from left to right: 50 μmol/L PD98059, 30 min; control; pretreatment with 50 μmol/L PD98059, 30 min, then intervened with 0.1 mg/L BER 15 min; 0.1 mg/L BER for 15 min) were examined using western blot. (*vs* control, *P < 0.05; **P < 0.01, ***P < 0.001; *vs* BER, ^◆^P < 0.05) (GEF: Gefitinib, BER: Berberine).

The effects of BER on hPDLSC were observed after treatment with EGFR-specific receptor inhibitor GEF and ERK pathway specific inhibitor PD98059. As shown in Fig. 5A, compared with the control group, the protein levels of p-EGFR, p-ERK and FOS were decreased in the GEF (2 μg/L, 24 h) treatment group, but increased in the 0.1 mg/L BER group (15 min) and GEF + BER (pretreated with 2 μg/L GEF group for 24 h, then intervened with the 0.1 mg/L BER for 15 min), while the levels in the group of GEF + BER, were decreasing compared with the BER group. In Fig. 5B, the protein levels of p-ERK and FOS declined in 50 μmol/L PD98059 treated for 30 min, and rose in the BER treatment group (0.1 mg/L 15 min) and PD + BER group (pretreated with 50 μmol/L PD98059, then intervened with the 0.1 mg/L BER for 15 min) when compared with the control group. Meanwhile, the expression levels for p-ERK and FOS in the group of PD + BER decreased when compared with the BER group. However, p-EGFR did not change significantly in the PD98059 treatment group, but rose in the group of BER treated alone, in contrast with the control group. At the same time, compared with the BER group, the level of p-EGFR showed no significant change in the group pretreated with PD98059, then intervened with BER. These results indicate that the ERK pathway inhibitor PD98059 had no effect on the protein expression of the upstream EGFR.

## Discussion

**Figure 6:**
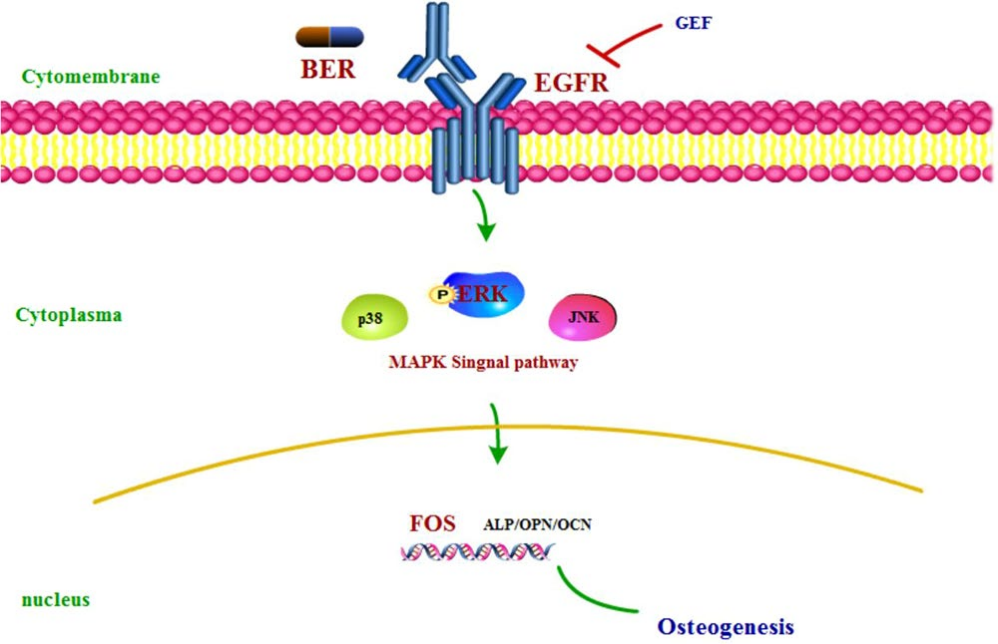
summary.

Through this research, we have obtained the following results: BER could bind to EGFR on the cell membrane of hPDLSC; the intracellular ERK signalling pathway was subsequently activated; the nuclear-related genes FOS up-regulated; and the osteogenesis effect of hPDLSC accelerated (Fig. 6).

Chronic periodontitis (CP) is a bacterial infectious disease with high prevalence in the world. With the development of the disease, some clinical symptoms can gradually appear, eventually leading to the damage of periodontal tissue. hPDLSC play an important role in the process of periodontal bone regeneration. Current periodontal treatments have been inseparable from the auxiliary role of drugs. TCM has a variety of biological effects, such as promoting cytoplasmic proliferative activity and osteogenesis, so it is very suitable for chronic periodontitis – a chronic disease caused by bacteria. Preliminary research through CMC indicates that there was active binding between *Coptidis Rhizoma* and the membrane receptor of hPDLSC, and that Berberine (BER) is the active ingredient from *Coptidis Rhizoma*^7^. The literature reviews^12–14^ and Fig. 1 both indicate that BER has the effect of regulating bone balance of hPDLSC, such as promoting cell proliferation and increasing osteogenesis. These effects of the drug are very suitable for the treatment of chronic periodontitis, especially in promoting the regeneration of periodontal bone tissue. Which membrane receptor was responsible for the specific affinity between BER and hPDLSC, and the molecular signal transmission and signalling pathways in achieving the promotion of the periodontal bone tissue repair mechanism was not clear; this was the focus of the current study. The effects of BER on osteogenesis were reported in 2008^8^. However, in our research, we not only confirmed that BER could promote the osteogenic effect of hPDLSC, but also explored that the effect of active binding between BER and the membrane surface of hPDLSC through the membrane receptor of EGFR, which further leads to the activation of intracellular ERK signaling, promoted the enhancement of the intracellular gene FOS. Therefore, although the osteogenic effect of BER has been reported in previous studies, we further investigated the membrane surface targets of cell activity between drugs and hPDLSC, which could provide an experimental basis for further drug design and synthesis and lead to more effective drugs that aim at this drug target.

CMC, a new affinity chromatography method developed in recent years, can be used to study the role of drug ligands and receptors. In this study, we found that the activity binding target on the surface of hPDLSC with BER may be EGFR, through the retention on CMC, competitive binding experiment and K_D_. As a new screening method, the CMC only illustrates that there is a strong combination between the drug and the receptor. Whether the effect of regulation is positive or negative needs further validation in biological experiments; therefore, further experiments in this study showed that BER, through EGFR on hPDLSC, could promote cell proliferation and the osteogenesis effect. BER may cause intracellular positive regulatory effects through activating EGFR and ERK (whereas GEF and PD98059 respectively produce negative regulatory effects as a specific inhibitor for the EGFR target and ERK signal pathway). The effects of promoting proliferation and osteogenesis of BER on hPDLSC is a promising application for periodontal bone defects caused by chronic periodontitis, so it could be used as a new drug for the treatment of chronic periodontitis.

It has been reported in the literature that the MAPK signalling pathway was important in pharmacological studies of BER and for osteoblastic differentiation of hPDLSC^8,9^. MAPK has also been widely recognised as a downstream signalling pathway upregulating specific genes, thereby facilitating osteogenic differentiation of rBMSCs^15,16^. Thus, for a better understanding of the intracellular osteogenic mechanism, the MAPK signalling pathway was studied by determining its downstream ERK, P38, and JNK signalling pathways using Western blot analysis. The intracellular signal transduction pathway associated with ERK is thought to be a classical MAPKs signal transduction pathway. The MAPK/ERK signalling pathway plays an important role in osteogenesis and has become a hotspot for recent research^17–20^. As an important receptor, tyrosine kinase, EGFR could combine with EGF on the membrane, activate the phosphorylation cascade (EGF/EGFR → Ras → Raf → MAPK/ERK), and cause the changes to transcription factors and proteins in the nucleus, eventually having a variety of biological functions^21^. Therefore, we can assume that BER may occur through the membrane receptor EGFR-mediated intracellular ERK pathway, causing the upregulation of the target gene FOS.

The epithelial growth factor receptor (EGFR) was found to be a possible membrane receptor. In fact, this receptor is one of the important transmembrane receptors for the MAPK signal. It was firstly reported in 1963 that EGF/EGFR can promote corneal healing^22^, and it was later found to have a certain role in cell viability, migration, proliferation, anti-inflammatory, wound healing and promoting bone repair^23–27^. The location of EGFR in periodontal tissues reported that this receptor was highly expressed in periodontal ligament cells, pre-osteoblasts and vascular cells. This result suggested that EGFR could play a significant role in the regeneration of periodontal tissue^28^. On the other hand, the damage of periodontal tissue by *porphyromonas gingivalis* could be performed by deactivate EGF/EGFR^29^.

It was reported in the literature^30^ that BER could inhibit colitis-associated tumorigenesis, by suppressing inflammatory responses and via EGFR signalling-involved tumor cell growth at a concentration of 25 μmol/L. In our study, we found that BER could promote the effect of osteogenesis in 0.1 mg/L (0.2 μmol/L). This result suggested that a drug made of BER could have different, even opposite, biological effects in different concentrations. Simultaneously, this study has shown that BER could have a biological effect through EGFR. In our study, we used CMC to screen out that EGFR might be the target site between BER and the membranes of hPDLSC, and that BER could play a positive role in cell growth and osteogenesis at the appropriate concentration. This finding is important both to find the target site between the drugs and cells related to some diseases, and to explore the specific mechanism of the drugs.

It has been shown that *Coptidis Rhizoma* and its active ingredient BER can control the inflammation of periodontitis in the treatment of clinical periodontitis, promote the proliferation and osteogenesis of hPDLSC, and achieve the purpose of repairing periodontal bone damage. The screening system of CMC confirmed that BER did indeed bind to hPDLSC actively. The results of this study will allow the exploration of the effective drugs for chronic periodontitis to be viewed from a new perspective. The further structure optimization and adjustment from the aspect of the membrane receptor, can allow the drug to cause better pharmacological effects and have lower toxic side effects. Therefore, it is worthy of further in-depth study.

## Materials and Methods

### Chemicals and Materials

Berberine (BER), Gefitinib (GEF), Tan roroxin, Metoprolol tartrate (N98% pure) and other receptor inhibitors were purchased from the National Institutes for Food and Drug Control of China (Beijing, China). The reference sample has been deposited at the Specimen Laboratory, Research and Engineering Center for Natural Medicine, Xi’an Jiaotong University (Xi’an, China). Silica gel (ZEX-II, 5 μm, 200 Å) was obtained from Qingdao Meigao Chemical Co., Ltd. (Qingdao, China). HPLC-grade methanol was acquired from Burdick & Jackson (NJ, USA). All other reagents used were of analytical grade. Stock solutions for analysis (1 mg/mL) were prepared by separately dissolving the standard drugs in methanol. Standard solutions (0.01 mg/mL) were prepared by dilution of each stock solution with mobile phase (ultrapure water). hPDLSCs were cultured in the Research Center, College of Stomatology, Xi’an Jiaotong University. Dulbecco’s Modified Eagle Medium/F12 (DMEM/F12), fetal calf serum (FCS) and trypsin were purchased from Sigma (Saint Louis, MO, USA). Type I collagenase (Worthington Biochem, Freehold, NJ, USA), ascorbic acid 2-phosphate (Wako, Tokyo, Japan), glutamine, penicillin and streptomycin were also used in the study, PD98059 (Med chem express).

### Preparation of standard solutions

Standard stock solutions of Berberine (BER), Gefitinib (GEF) and so on (1 mg/mL each) were separately prepared in methanol; standard solutions (0.01 mg/mL) were prepared in 5 mmol/L ammonium acetate water solutions.

BER and GEF were diluted in dimethylsulfoxide (DMSO) stock solution prior to addition to the cell culture medium to form a mixture with a final DMSO concentration of <0.1% (v/v).

### Cell culture of hPDLSC and preparation for the hPDLSC/CMC system

According to the literature^7^, hPDLSC cultured in primary, immunocytochemistry identified for keratin and vimentin gave negative and positive results, respectively, to confirm that the cells were hPDLSC, and thus, could be used for subsequent experiments. The cell suspensions were seeded into dishes with DMEM/F12 medium supplemented with 10% FCS, 100 μmol/L ascorbic acid 2-phosphate, 2 mmol/L glutamine, 100 U/mL penicillin, and 100 μg/mL streptomycin, and then incubated at 37 °C in a humidified atmosphere containing 5% carbon dioxide. Cells were sub-cultured every three days at a dilution of 1:3 using a 0.25% aqueous solution of trypsin and then resuspended into a single-cell suspension.

Cells from exponentially growing cultures were used in all experiments. A cell count was performed to determine the cell density. The cell density was ≥1 × 10^7^. The cells were washed three times with phosphate-buffered saline (PBS). A Tris-HCl hypotonic solution (50 mmol/L, pH 7.4) was added to the washed cells to produce the signal cell suspension. The cells were subsequently ruptured using ultrasonication for 30 min and the resulting homogenate was put in a centrifuge at 1000 g for 10 min. The pellet was discarded and the supernatant centrifuged at 12,000 g for 20 min at 4 °C. The resulting precipitate was resuspended in 10 mL Tris-HCl and the suspension was re-centrifuged at 12,000 g. A cell membrane suspension was obtained by adding 5 mL of PBS to the precipitate and stored until use. According to a previously reported method^31^, 0.05 g silica was activated at 105 °C for 30 min and used as a carrier; it was then homogenised with the cell membrane suspension by adding the mixture slowly to it under a vacuum and agitated at 4 °C to obtain the hPDLSC membrane stationary phase (CMSP). The CMSP was then packed into a column (10 × 2.0 mm i.d.) through a wet-packing procedure to yield the hPDLSC/CMC column. Ultrapure water, which was delivered at a flow rate of 0.2 mL/min, was used as the mobile phase in the CMC system. The detection wavelengths were 345 nm (BER), 245 nm (GEF) and 397 nm (CAP), and the column temperature was 37 °C.

### Application of the hPDLSC/CMC system

Any retention fraction which was “recognised” by the hPDLSC/CMC model was captured. By using BER as a comparison, this method was used to screen the situations of the activity combination form of BER, GEF, CAP, which had the positive or negative chromatographic peaks toward hPDLSC membrane receptors.

### Competitive displacement assay

In order to verify whether BER was active on the same site of hPDLSC membrane receptors with GEF or other receptor inhibitors, we performed a competitive displacement test. K_A_ values in the hPDLSC/CMC system were measured using a series of Berberine solutions (0 to 16 × 10^−7^ mol/L) in the mobile phase. The chromatographic parameters were the same as above. The retention times of BER at different concentrations were recorded. Next, standard solutions of BER and GEF were injected into the hPDLSC/CMC column. The capacity factor (*k*′) of the CMC chromatographic peak in the elution curve was calculated using Eq. (1):

1 $$k^{\prime} =({t}_{{\rm{R}}}-{t}_{0})/{t}_{0},$$

where *t*_R_ is the retention time of the ligand and *t*_0_ is the dead time of non-retained solvent. The values of K_A_ and K_L_ could be calculated from Eq. (2):

2 $$\frac{1}{k^{\prime} -X}=\frac{{K}_{A}{[R]}_{S}}{{K}_{L}{V}_{M}}{[L]}_{m}+\frac{{K}_{A}{[R]}_{S}}{{V}_{M}}$$

where K_A_ and K_L_ are the equilibrium dissociation constants for the analyte and marker in the mobile phase, respectively; [L]m, and [R]_S_ are the molar concentrations of the ligands in the effluent and immobilised receptors at the surface of the stationary phase, respectively; and V_M_ is the dead volume of the column. The term X was introduced to the denominator with the *k*′ value to eliminate errors incurred during iterative testing. When Berberine is used both as a marker and an analyte, K_A_ and K_L_ (which was obtained from the ratio of intercept and slope, respectively) are equal. [R]_S_/V_M_ is equal to the slope. Therefore, upon substituting Eq. (2) in Eq. (3) the following is obtained:

3 $$\frac{1}{k^{\prime} }=\frac{{[R]}_{S}}{{V}_{M}}{[L]}_{m}+\frac{{K}_{A}{[R]}_{S}}{{V}_{M}}$$

The K_A_ values of the two ligands were obtained using linear regression of the plot of 1/*k*′ versus [L]m.

### Molecular docking

Molecular docking consists of three steps: definition of the structure of target molecule (EGFR: 2ITY); location of the binding site; and determination of the binding mode. The binding score of BER and GEF with the membrane receptors EGFR can then be obtained.

### PCR Array

A PCR array combines the characteristics of sensitivity, specificity of qPCR and the high throughput of the chip, which could greatly shorten the experimental cycle and be an ideal way to study the expression level of pathway-specific genes. The steps are similar to Real-time PCR, including preparing cDNAs from RNA samples, adding cDNA to a Real-time PCR Master Mix, aliquoting the Mixture across the MAPK PCR Arrays and performing thermal cycling, then analysing fold changes in expression.

### Real-time PCR

Total RNA was extracted using Trizol reagent according to the protocol. Reverse transcription (RT) was performed with 2 μg total RNA using M-MLV reverse transcriptase to synthesise first-strand cDNA, following by cDNA amplification using the specific primers for GAPDH, and ALP, OPN, OCN (Table 1).

**Table 1:** Real time PCR primers.

gene	primers (5′-3′)	products (bp)
GAPDH	(F) ATCGTGCGTGACATTAAGGAGAAG	179
	(R) AGGAAGGAAGGCTGGAAGAGTG	
ALP	(F) CATGCTGAGTGACACAGACAAGAA	141
	(R) ACAGCAGACTGCGCCTGGTA	
OPN	(F) ACACATATGATGGCCGAGGTGA	116
	(R) GTGTGAGGTGATGTCCTCGTCTGTA	
OCN	(F) CCCAGGCGCTACCTGTATCAA	159
	(R) GGTCAGCCAACTCTGCACAGTC	

### Western blot

Cells were lysed in RIPA lysis buffer, and the lysates were harvested by centrifugation (13,523 g) at 4 °C for 30 min. Approximately 30 μg protein samples were then separated by electrophoresis in a 12% sodium dodecyl sulfate polyacrylamide gel and transferred onto a polyvinylidene fluoride membrane. After blocking the nonspecific binding sites for 60 min with 5% non-fat milk, the membranes were incubated overnight at 4 °C with a mouse monoclonal antibody against GAPDH, ALP, OPN, OCN, Anti- EGFR, p-EGFR, Anti-ERK, p-ERK, AKT, p-AKT, p38, p-P38, FOS (Table 2). The membranes were then washed three times with TBST for 10 min and probed with the horseradish peroxidase (HRP) IgG antibody at 37 °C for 1 h. After three washes, the membranes were developed by an enhanced chemiluminescence system.

**Table 2:** Western blot antibodies.

protein	First antibody	Secondary antibody
GAPDH	1:500 (Rabbit anti-human)	1:20000 (Goat anti-rabbit)
ALP	1:200 (Mouse anti-human)	1:20000 (Goat anti- Mouse)
OPN	1:100 (Goat anti-human)	1:40000 (Rabbit anti- Goat)
OCN	1:1000 (Mouse anti-human)	1:30000 (Goat anti- Mouse)
EGFR	1:1000 (Rabbit anti-human)	1:20000 (Goat anti-rabbit)
p-EGFR	1:1000 (Rabbit anti-human)	1:20000 (Goat anti-rabbit)
FOS	1:500 (Goat anti-human)	1:40000 (Rabbit anti- Goat)
ERK	1:1000 (Mouse anti-human)	1:20000 (Goat anti- Mouse)
p-ERK	1:1000 (Mouse anti-human)	1:20000 (Goat anti- Mouse)
P38	1:1000 (Mouse anti-human)	1:20000 (Goat anti- Mouse)
p-P38	1:1000 (Mouse anti-human)	1:20000 (Goat anti- Mouse)
JNK	1:1000 (Mouse anti-human)	1:20000 (Goat anti- Mouse)
p-JNK	1:1000 (Mouse anti-human)	1:20000 (Goat anti- Mouse)

### Statistical analyses

All experiments were repeated three times. Statistical analysis was performed by using SPSS18.0. Data were evaluated through one-way analysis of variance (ANOVA) followed by Tukey’s test as a post hoc comparison. All data are presented as mean ± standard error of the mean (SEM). The probability level (P) was considered significant at P < 0.05.
